# On the Functions of the Great Sympathetic Nerve, or the Nerve of Organic Life

**Published:** 1852-07

**Authors:** William Gries

**Affiliations:** The city of Reading, Pennsylvania


### MEDICAL EXAMINER,

AND

### RECORD OF MEDICAL SCIENCE.

NEW SERIES.—NO. XCI.—JULY, 1852.

### ORIGINAL COMMUNICATIONS.

*On the Functions of the Great Sympathetic Nerve, or the Nerve of Organic Life.* By Wm. Gries, M. D., of the city of Reading, Pennsylvania.

In an essay, which I had the honor of reading before the "Medical Society of the city of Reading, and county of Berks," on the 6th of April last, and again before the "Medical Society of the State of Pennsylvania," at its annual session in the last week in May, at Philadelphia, I made the following cursory remark: "Upon serious reflection I looked upon all the diseases as being under the influence of some cause affecting more particularly the organic nervous system, prostrating it, and disturbing its proper action—and I concluded that this must be corrected—and it appeared to me that the sulphate of quinine, in proper doses, would do this better than any other medicine." If I am not deficient in information, the opinion held by the profession in general, with reference to the functions of the organic nerve, is, that it may be compared to lines of telegraphic wires extended from one organism to another, to give mutual information how each performs its functions, and to combine all the organisms in a concert of action, to work out the great objects of manifesting life. This I have been led to consider a very imperfect and partial view: and I have been perfectly astounded that the views brought out, more than thirty years ago, by Dr. Jas. Copland, of England, and so ably extended and maintained in his great work, "the Dictionary of Practical Medicine," should have attracted so little attention from the profession. The more I have studied the subject in his writings and by my own observations upon the diseases that have come under my notice, the more have I been convinced of their truthfulness; and I refer with confidence to the cases recorded in my essay above referred to, as illustrative and confirmatory of those views. Whilst I was making those observations, I was perfectly electrified when I read, in the April number of the "American Journal of the Medical Sciences" the experiments performed on the great sympathetic nerve, by M. Claude Bernard; and before I had finished the reading of that short notice, I was irresistibly convinced that it fully confirmed all those views of Dr. Copland. I reasoned thus: if the destruction of a small cervical branch of the sympathetic has the effect of producing those results stated in that notice, it must, of necessity, follow, that an impairment of its vitality must, in a degree commensurate with the degree of that impairment, be followed by similar results on the whole system. I think that I may safely say, that I have now treated more than one hundred cases, similar to those recorded in my essay above referred to, upon the same plan, and I hesitate not to say, that, without a single exception, with equal success. In the observations I have made upon those cases I have much extended Copland's views, as far as their record has come under my review. I now look upon the great sympathetic as God's prime minister, appointed to preside over the whole organization of man, and to actuate and regulate all its vital actions, both physical and mental.

I believe that the nervous system of animal life, is as much under its influence and guidance for the performance of its proper functions, as any other organism in the body. In further illustration of my views I am tempted to say, with trembling awe, fearing that it borders on blasphemy, that the combined nervous systems are a triune government; that the great sympathetic operates through the system of animal life, and emanating from both, the nervous fluid, or whatever name I may give it, manifests its power and action on the organism.

In illustration and confirmation of my views, which I have so categorically stated, I shall now recite other cases, and make observations upon them. As I have been myself the chief subject of experiment, I must now, even at the risk of being accused of egotism, give something of my personal history. However, I feel confident that the true physiological enquirer, for whom alone I write, will clear me of such an imputation.

About forty six years ago, when I was ten years of age, I had the intermittent fever for three months, of a tertian form. I could not retain sufficient cinchona on my stomach to interrupt or put a stop to the paroxysms, and my physician gave me frequent emetics without attaining that end; and I began to hate him and his emetics; for the sight of him was almost enough to make me vomit. I remember well yet, that in the latter part of my sufferings, when the paroxysm was at its height, I would have a sensation as if my head was expanding, and at last filling the whole house that I was in; when, of a sudden, I would be transported to the edge of a precipice or a chasm, and in the act of toppling over, I would scream out in agony; and my father would lead me or carry me about, which seemed to relieve me. From that time onward, all my mental faculties seemed to be blunted. My memory was less retentive; I was stupid and drowsy. I would frequently, when a grown up young man, fall asleep in the midst of company, even that of young ladies. I tried various plans to counteract it—low diet, &c.—but all without effect. Notwithstanding this impairment of my mental faculties, I never lost my appetite for mental food. I was mentally omnivorous. I thought frequently of Bacon's Aphorism, "reading makes a full man, writing a correct man, and conversation a ready man," and I often resolved to be governed by it, but never succeeded. I would add to the first branch—it also makes a dull and stupid man—for repletion with mental food has the same stupifying effect, that repletion has upon our physical organization. That I never benefitted by the second is abundantly evident, from the specimen before the reader. I have no power for precision in language; consequently I employ figures or analogies for illustration. I take hold of a figure, but I don't carry it out, and as

I pass on I fling away one and grasp at another, as it comes readily to hand. I have written but little, and, with the unfaithful steward, I buried my one talent, and took credit to myself for doing so. With reference to the last branch, I had but little opportunity to benefit myself, until the formation of the American Medical Association, and its auxiliaries.

I tried various means to excite my mental faculties; all alcoholic stimulants, distilled or fermented, always rendered me more stupid. When phrenology came in vogue, I paid considerable attention to the subject, and came to the conclusion that my brain, being a very large one, was made of very coarse material, and was never intended for literary or scientific occupation; and the rest of my physical structure being of little strength, I was led to fear that I was but poorly fitted for any station. As regards the phrenological development of some of the organs, veneration is very deficient, so that it requires a continual effort to properly venerate the Author of my being; but firmness, or what in me might, perhaps, more correctly be called stubbornness, is very strongly developed. Now the subject is described; next come the experiments.

At an early period of the prevalence of the peculiar state of disease described in the essay referred to above, I found my health deteriorating; my hepatic secretions and my bowels were particularly affected; it seemed paroxysmal, generally worse late in the afternoon and night. On account of being, for many years, a sufferer from emphysema of the lungs, I feared the use of quinia in large doses. I used mercurial alteratives and mild laxatives, to mend the secretions, but they would not be mended. My appetite was impaired, my strength failed, and I was unapt for mental or physical exertion. I determined at length, by observing the benefit resulting on others from large doses of quinine, to use it on myself. I commenced by taking ten grains in the evening, before going to bed; it was followed, in about one hour, by a slight tremor in my hands, and in fact in all the voluntary muscles; but what completely and agreeably surprised me, and what I had not, in the smallest degree, anticipated, was the clearness and expansion of my mental faculties; I could think more clearly; and, after the lapse of about two hours, I fell asleep; and slept more soundly than I had for many years—(for although I was always drowsy more or less, unless engaged in bodily exercise, yet I never slept soundly, and my awakening was not sudden.) I felt much better in the morning, more inclined to attend to my duties, and more capable of attention.

Upon reflection, through the day, I concluded that I had taken an overcharge (like an overcharge of an electric battery,) and I took a diminished dose in the evening. It was followed by less tremor, but equally sound sleep and readiness for mental and bodily action. In a few days I brought the dose to five grains, and took it before breakfast in the morning: at this dose it produced no tremor, and was followed by all the good desired. I have been taking it regularly now for four months, with an occasional interruption of two or three days. My appetite has been good, my secretions are corrected, my mind and body are invigorated; I have had no occasion for alteratives or purgatives. In a word, I have enjoyed life more for the last four months, than I had done for forty-six years. I had for a considerable time held the view that the remote cause of ague operated upon the great sympathetic, impairing, in some manner, its functions, and that this impairment was the proximate cause of ague; now I feel perfectly convinced of its truth, and, in my own case, reason: that my protracted ague, at ten years of age, impaired the power of the sympathetic, and that the sulph. quiniæ has restored that power. I further believe that that terrific disease, tetanus, may reasonably be ascribed to the impairment of the superintending power of the sympathetic over the nervous system of animal life, and that the latter runs furiously wild without the controlling check of the former. And I further believe that in very large doses of quinia, frequently repeated, will be found the remedy for this opprobrium of medicine. Nay, I go still further, to believe that even hydrophobia will yet submit to it. I feel that I am riding a hobby, and I am terrified when I reflect that many men of much superior capacity and experience have been engulfed in the slough and quagmire, by not restraining their hobbies; yet I cannot find prudence enough to draw in the reins of mine; his gait is so pleasant, I cannot look down, but up, and under a charm I go ahead. I write this only, as it were, to call for help, that if my friends see my danger they may cry out and stop me.

I hope that no one will understand me to say, that the sulph. quin. is a remedy for all kinds of morbid action in the sympathetic. It is intended only in its dynamic action. It is only the remedy when it is in an adynamic state, to raise it to its proper standard, and to re-establish it in its proper office. Thus far my experience warrants me. That other morbid states may arise requiring different therapeutic agents, I have no doubt; but I do not understand them. My main object is to draw attention to its functions and to excite inquiry.

I have found it necessary to use more tonics of all sorts during the last six months than I did during ten years before, in proportion to the number of patients. Indeed, twenty-five years ago I was inexperienced enough to believe, that tonics might be expunged from the list of therapeutic agents. In order to show the perfect safety of the large doses of quinine, spoken of in my former essay, I will state, rather minutely, the case of a little girl, now two years of age. She was from birth of a delicate constitution, always very restless. About six months ago, when I was called to see her, she had pretty well developed symptoms of inflammation of the brain:—I got her over it with difficulty, and she had a protracted convalescence. During an early period of the epidemic scarlatina of last winter, she was attacked by that disease, and had a slow convalescence. About six weeks ago, she was taken with a severe attack of measles.

I ought to have mentioned that she was, almost from birth, affected by a slight cough. After the measles passed off, she was weak, fretful and sleepless. I had to give her opiates, and then again alteratives, to do away the mischief that the opium had done. I thought in her convalescence from scarlet fever that she was affected by the general epidemic influence, and I gave her, several times, two grain doses of quinia, with benefit.

During the protracted recovery from measles, I gave, on the 21st of May, four grains of sulph. quiniæ, in the evening; next morning the mother reported, that she had slept better that night, than she had for many nights before, and awoke much more cheerful. I gave her again four grains of quinia, and the next morning her mother remarked, when I called to see her, "Doctor, it is very strange that Amelia, always, when she takes that white medicine, sleeps better than on anything else, and awakes more cheerful. On the 24th of May, as I was preparing to go to Philadelphia, I left a mixture of four grains of sulph. quin. to be given on the 28th, to prevent a relapse. Last evening her mother sent for some drops, to make her sleep, and I sent the four grain quinia mixture ; this morning (June 4th,) I found she had slept well on it, and was very cheerful. I think it not out of place here to state another very remarkable case. On the 10th of April, I was called to a lady, who had born, three children, at single births, who are all living. She had been sick five days, under the hands of another *doctor.* I found her vomiting blood largely; she had great tenderness over the epigastrium—a costive state of bowels. She had also occasional bleeding from the nose. She thought that she might be two months enceinte. Her tongue, when protruded, was contracted, pointed, and of a deep red. Her *doctor* had given her laudanum and ether for her pain ! and hot strong cinnamon tea to stop vomiting ! According to her account she had an hemorrahagic diathesis from her youth up.

I commenced my treatment by interdicting all that had been done ; and ordered cups to thê epigastrium, and an enema of turpentine and castor oil. I directed them to saturate lemon juice with table salt, and to put a teaspoonful of this into half a pint of iced water, and to use this altogether for her drink. She immediately improved on this ; but the case proved exceedingly protracted and intractable. On the 1st of May, I tried nitr. argenti with opium, but I had soon to desist, for it irritated the stomach. I then tried creasote in small doses, but it would not answer. On the fourth of May I determined to use the sulph. quiniæ, because I had observed for several days a paroxysm every afternoon, manifesting itself by a little coldness of the knees, and, crampy pain of the stomach, with distressing nausea and vomiting of blood ; but her stomach would not tolerate the quinine. I then made up my mind to introduce the quinine, in large doses, into the rectum on the sixth of May. But early in the morning her husband came into my office, to tell me that she insisted upon having a homœopathic doctor. He attended her three days with great promises but no performance ; when they applied to one of our old physicians, of the old school. On the 15th of May, they begged of me to take the case again in hand. Upon visiting her I

found her exceedingly prostrated and emaciated. I immediately proceeded to carry out my project; and from the 15th to the 21st of May, I had administered eighty grains of sulph. quiniæ, by enemata. On the 24th of May I left her fairly convalescent. I was absent nine days, and on the day before my return, she had a slight relapse; of this she is now cured by twenty grains of quinine, by enema.

